# Pre-surgical connectome features predict IDH status in diffuse gliomas

**DOI:** 10.18632/oncotarget.27301

**Published:** 2019-11-05

**Authors:** Shelli R. Kesler, Rebecca A. Harrison, Melissa L. Petersen, Vikram Rao, Hannah Dyson, Kristin Alfaro-Munoz, Shiao-Pei Weathers, John de Groot

**Affiliations:** ^1^Cancer Neuroscience Laboratory, School of Nursing, The University of Texas at Austin, Austin, Texas, USA; ^2^Department of Diagnostic Medicine, Dell School of Medicine, The University of Texas at Austin, Austin, Texas, USA; ^3^Department of Neuro-Oncology, The University of Texas MD Anderson Cancer Center, Houston, Texas, USA; ^*^These authors contributed equally to this work

**Keywords:** MRI, IDH, glioma, machine learning, connectomics

## Abstract

**Background:**

Gliomas are the most common type of malignant brain tumor. Clinical outcomes depend on many factors including tumor molecular characteristics. Mutation of the isocitrate dehydrogenase (IDH) gene confers significant benefits in terms of survival and quality of life. Preoperative determination of IDH genotype can facilitate surgical planning, allow for novel clinical trial designs, and assist clinical counseling surrounding the individual patient’s disease.

**Methods:**

In this study, we aimed to evaluate a novel approach for non-invasively predicting IDH status from conventional MRI via connectomics, a whole-brain network-based technique. We retrospectively extracted 93 connectome features from the preoperative, T1-weighted MRI data of 234 adult patients (148 IDH mutated) and evaluated the performance of four common machine learning models to predict IDH genotype.

**Results:**

Area under the curve (AUC) of the receiver operator characteristic were 0.76 to 0.94 with random forest (RF) showing significantly higher performance (*p* < 0.01) than other algorithms. Feature selection schemes and the addition of age and tumor location did not change RF performance.

**Conclusions:**

Our findings suggest that connectomics is a feasible approach for preoperatively predicting IDH genotype in patients with gliomas. Our results support prior evidence that RF is an ideal machine learning method for this area of research. Additionally, connectomics provides unique insights regarding potential mechanisms of tumor genotype on large-scale brain network organization.

## Introduction

Gliomas originate in the brain and are the most common type of malignant primary brain tumor. High grade gliomas are comprised of histologic grade III (anaplastic astrocytoma, and anaplastic oligodendroglioma) and grade IV (glioblastoma multiforme) tumors and account for the majority of diffuse gliomas. Despite being histologic grade II, low-grade gliomas eventually progress and transform to a higher grade. Therefore, it is increasingly appreciated that histologic grade alone does not account for the variability in outcome among patients with these cancers. In fact, disease prognosis depends on multiple factors including both clinical and molecular features of the tumor.

Patients with mutation of the isocitrate dehydrogenase (IDH) gene demonstrate markedly improved clinical outcomes compared to those with the wild-type tumor [1]. IDH status has been recognized to be of central biologic and prognostic import, and is now incorporated in the diagnostic deifinition of diffuse gliomas in the updated Word Health Organization diagnostic compendium for CNS tumors [2]. The presence of IDH mutation plays a significant role in response to treatment including extent of surgical resection [3] and chemoradiation [4]. Stratification of patients based on IDH status in certain clinical trials may be indicated [5]. Patients with wild type tumors also show lower cognitive function compared to the mutant variant [6]. Therefore, baseline prediction of IDH status is of great clinical importance for therapeutic decision making, including choice of therapeutic intervention such as IDH inhibitors and the decision to initiate treatment early. Additionally, presurgical knowledge of IDH genotype would be invaluable for risk stratification in clinical research and clinical counseling surrounding the individual patient’s disease.

IDH genotype is typically determined from biopsy or resection. However, several studies have demonstrated that conventional, pre-operative MRI can be used to non-invasively predict IDH status [7–10]. These radiomic approaches involve extracting relevant radiographic features believed to be associated with aspects of tumor phenotype. Multimodal imaging sequences are typically employed including FLAIR, T2, T1 pre-contrast, T1 post-contrast and DWI. Feature extraction focuses on tumor regions of interest. Other methods include MRS detection of 2-hydroxyglutarate accumulation in the tumor, which is associated with mutation [11].

However, our group and others have demonstrated that focal tumors are accompanied by widespread disruption of the entire brain [6, 12]. Importantly, we have shown a discriminable pattern of large-scale connectome organization associated with IDH status suggesting that brain networks reflect the molecular properties of the tumor [6]. The brain incorporates both biologic and environmental processes in a bidirectional manner, providing a uniquely parsimonious and sometimes more sensitive summary of key diagnostic and prognostic features. Brain network organization is highly associated with age, gender, education level and socioeconomic status [13–16] and reflects effects of cancer pathogenesis and treatment [17]. These are all known prognostic factors in diffuse glioma represented within whole-brain network organization.

Connectomes are graphs that model the brain as a network of nodes (regions) and edges (connections). Nodes typically reflect some discrete parcellation of cortical and subcortical processing units and can be defined microscopically to macroscopically. Edges are defined anatomically (e.g. measurable white matter pathway) and/or statistically (e.g. correlation between functional time series). Our group and others have demonstrated significant connectome disorganization in patients with diffuse glioma [6, 12]. One previous study showed that pre-surgical, whole brain connectome features were accurate predictors of high grade glioma survival [18] and another indicated that connectome properties were correlated with progression-free survival [12].

For this study, we aimed to use gray matter connectomes obtained from non-contrast, T1-weighted MRI scans. T1 MRI is routinely acquired pre-surgically as part of standard of care for patients with brain tumors. T1 MRI is used ubiquitously in neuroimaging research to measure brain volumes and there exist coordinated variations in gray matter volumes that make connectome construction possible [19]. These structural covariance networks are highly heritable and are believed to reflect underlying axonal connections as well as common neurodevelopmental and neuroplastic processes [19]. Robust alterations in gray matter connectomes are regularly observed in various neurologic syndromes [20]. As noted above, we demonstrated that gray matter connectomes can be used to distinguish between IDH variants of high grade glioma [6]. We hypothesized that pre-surgical gray matter connectome features would accurately predict IDH status in patients with diffuse glioma.

## Results

As shown in Table 1, the mean age of patients was 43.85 +/– 15.12 years and 62% of patients were male. All histologic grades of diffuse glioma were represented: grade II (43%), grade III (27%), and grade IV (30%). The majority were of astrocytic histology (73%), and over half were IDH mutant (63%). Glioma involvement of the frontal lobe was most common (50%) followed by temporal lobe (28%) and 14% of patients had a multifocal tumor. A minority (*N* = 41, 18%) had MGMT status available and only 21% had available preoperative KPS (within 3 days prior to surgery).

**Table 1:** Patient characteristics

	***N* = 234 **
IDH (Mutant)	148 (63%)
Age	Mean = 43.85 +/– 15.12 Range = 18–82
Sex (Male)	146 (62%)
Grade II	101 (43%)
Grade III	63 (27%)
Grade IV	70 (30%)
Oligodendroglioma	57 (24%)
Astrocytoma	170 (73%)
Oliogoastrocytoma	7 (3%)
Tumor Hemisphere (Left)	168 (72%)
Tumor Location (Primary)	
Frontal	117 (50%)
Insular	17 (7%)
Occipital	1 (.4%)
Parietal	34 (15%)
Temporal	65 (28%)
Multifocal Tumor	33 (14%)
MGMT Promoter Methylation (*N* = 41)	Positive: 24 (59%) Negative: 17 (41%)
KPS (*N* = 51)	100: 15 (29%) 90: 21 (41%) 80: 11 (22%) 70: 4 (8%)

**Figure 1:**
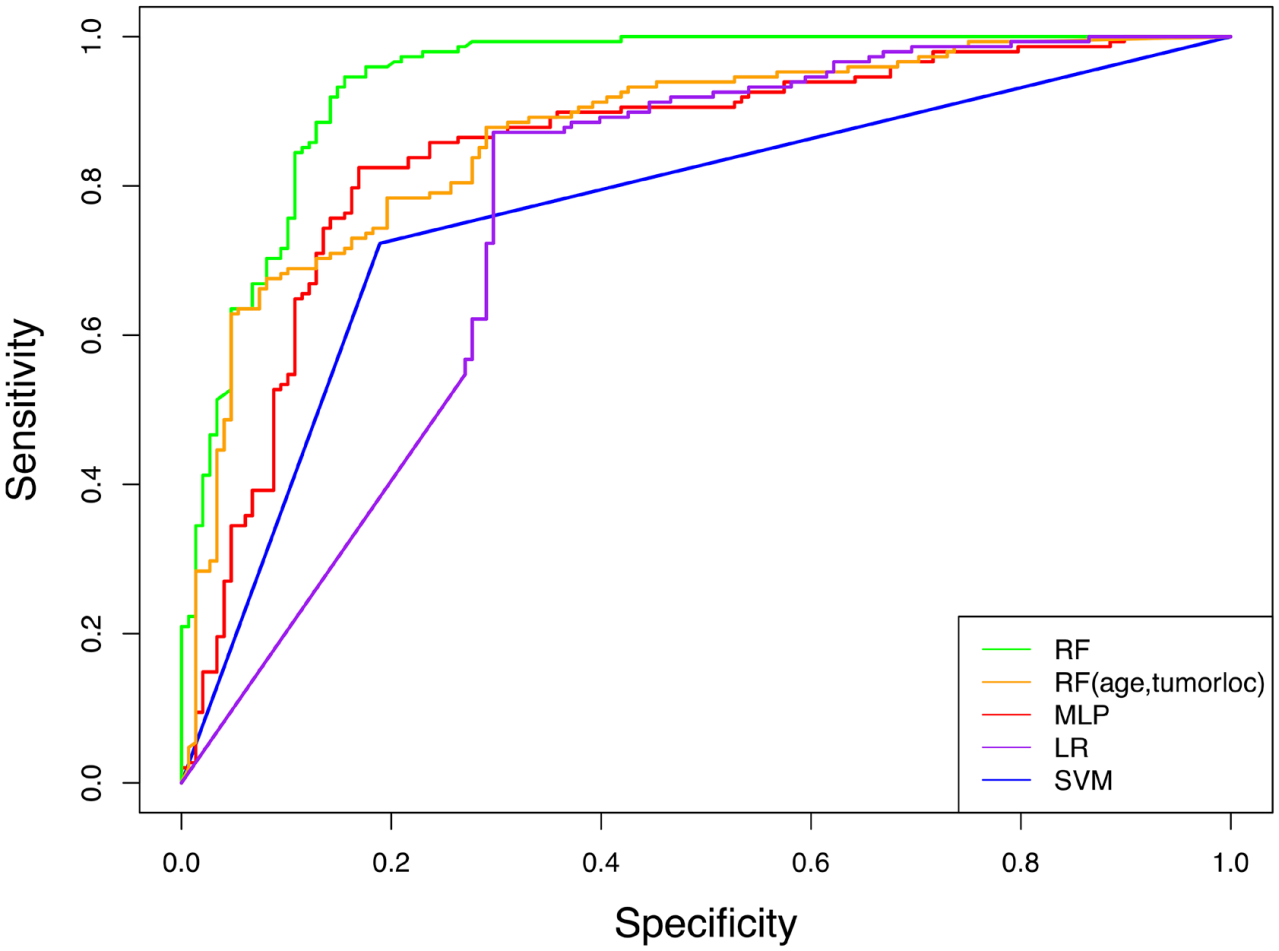
Receiver operator characteristic (ROC) curves for machine learning models predicting IDH genotype from connectome features. RF = random forest, MLP = multilayer perceptron, LR = logistic regression, SVM = support vector machine.

MLP and RF classifiers demonstrated the best performance in predicting IDH genotype with AUCs of 0.85 and 0.94, respectively. LR and SVM showed lower accuracies with AUCs of 0.76 and 0.77, respectively (Table 3, Figure 1).

**Table 2:** Machine learning approaches

Classifier	Description	Advantages	Tuning Parameters
Random Forest (RF) [44]	Ensemble of decision trees each trained on a random subset of features	Aggregates multiple independent classifiers, scale invariant, implicit feature selection, resistant to overfitting	ntree = 1000 mtry = 7 [log2(nfeats)+1]
Support Vector Machine (SVM) [45]	Defines an optimal hyperplane that maximizes the margin between classes	Kernel trick can solve complex problems, can handle imbalanced classes by weighting misclassification penalty	C = 1.0
Logistic Regression (LR) [46]	Multinomial logistic regression model with a ridge estimator	Simple, highly interpretable	ridge value = 1.0E-8
Multilayer Perceptron (MLP) [47]	Simple model of a biological brain that implements backpropagation	Can generalize in non-local ways akin to intelligent behavior, inherent feature selection	learning rate = 0.3 momentum = 0.2

**Table 3:** Machine learning model performance

Features	Model	Accuracy	Sensitivity	Specificity	AUC
90 connectome efficiencies, brain volume, network degree, network size	RF	86%	89%	83%	.94
	SVM	77%	79%	75%	.77
	LR	78%	84%	73%	.76
	MLP	80%	84%	77%	.85
90 connectome efficiencies, brain volume, network degree, network size, age, tumor hemisphere, tumor lobe	RF	89%	90%	89%	.95
Age, tumor hemisphere, tumor lobe	RF	77%	79%	76%	.87

RF = random forest, SVM = support vector machine, LR = logistic regression, MLP = multilayer perceptron, AUC = area under the curve.

The RF AUC was significantly higher than that of LR (*p* < 0.001), SVM (*p* < 0.001) and MLP (*p* = 0.012). MLP AUC was moderately higher than SVM (*p* = 0.042).

**Figure 2:**
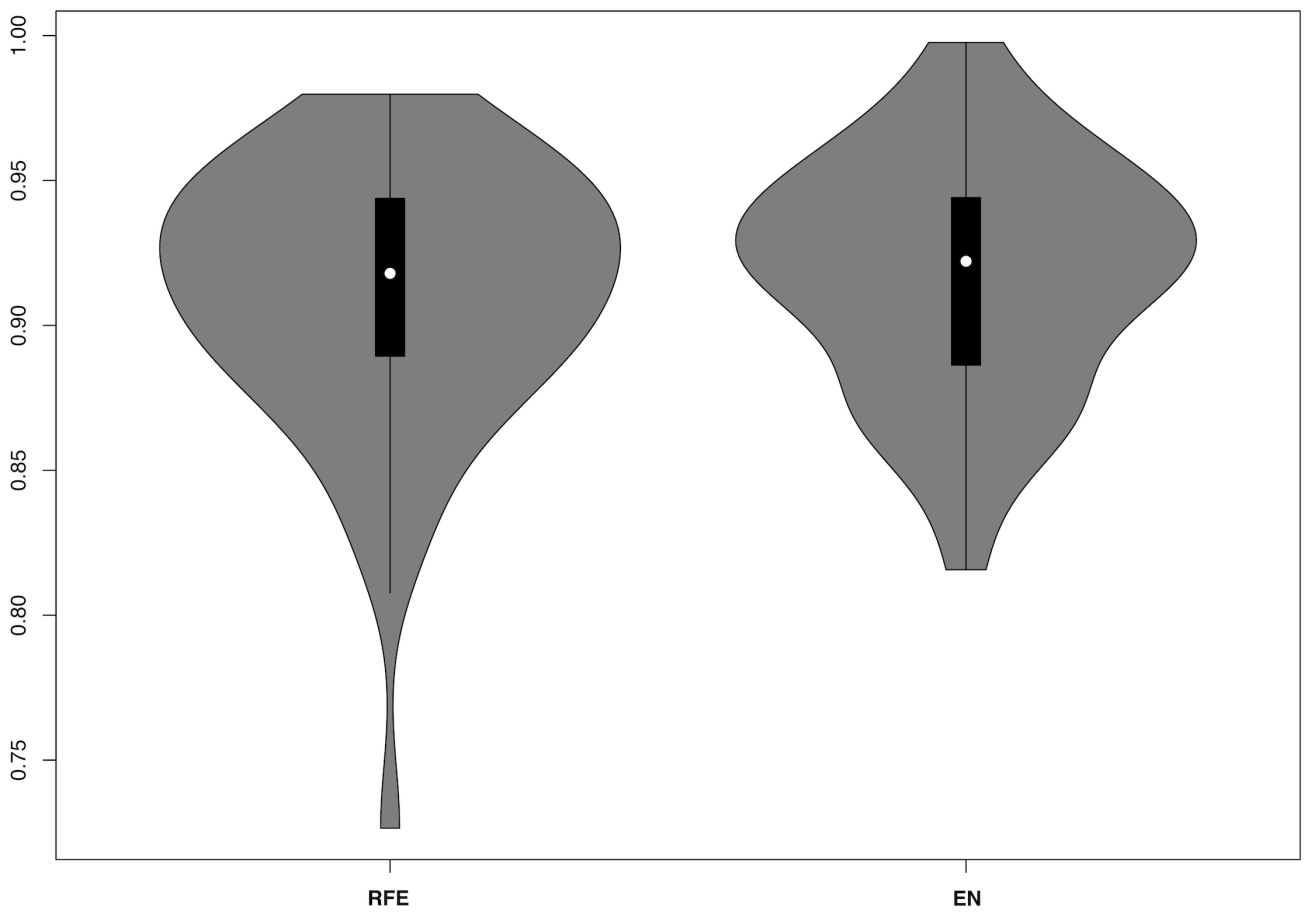
Violin plots for RF model AUCs including nested recursive feature elimination (RFE) or elastic net (EN) regression.

Feature selection did not seem to affect RF performance. Mean AUC for RFE was 0.91 +/– 0.05 and 0.92 +/– 0.04 for elastic net (Figure 2).

Adding age and tumor location to the RF model slightly increased accuracy (AUC = 0.95) but not significantly (*p* = 0.413). An RF model with only age and tumor location yielded an AUC of 0.87 (Table 3, Figure 1), which was significantly lower than the connectome RF model (*p* = 0.004).

## Discussion

We innovatively evaluated whole-brain connectome features with common machine learning algorithms to predict IDH genotype in 234 patients with diffuse glioma. Classifiers achieved AUCs of 0.76 to 0.94, with RF demonstrating the best performance. Our approach yielded results that were highly similar to those of radiomic methods. Zhang et al. reported an RF AUC of 0.92 for predicting IDH status from preoperative MRI radiomic features in 120 patients with grades III or IV glioma [8]. A study of 165 patients with low grade tumors showed an RF AUC of 0.79 [21]. Lu and colleagues reported higher accuracies (89–92%) for their RF radiomic models but in small, separate samples of high (*N* = 18) and low grade (*N* = 12) glioma [22]. Distinct from these studies, our method was applied across all histologic grades and phenotypes of diffuse gliomas. A recent radiomics study that also combined patients with low and high grade gliomas (*N* = 126) demonstrated a comparable RF AUC of 0.93 [10].

In addition to the equal or better performance of our models, our connectome approach presents potential advantages. Our method requires a single, standard of care, non-contrast MRI sequence that can be acquired in 5 minutes or less. Tumor segmentation required for radiomics feature extraction is typically manual or semi-manual which can be very time-consuming and complex. There are established, open source connectome software tools currently available [23] that could be integrated into clinical workflows and connectome measurement is computationally efficient. Connectome properties can provide insight regarding other factors important for clinical management of patients with glioma including cognitive outcomes. However, connectome features are affected by factors including choice of parcellation scheme, topological property and thresholding method, which should be carefully considered.

Our findings support the assertion by Wu et al. that RF be the preferred approach for predicting IDH genotype in patients with gliomas [10]. In addition to the advantages listed in Table 2, RF models are versatile, have low computational cost, involve simplistic implementation, are able to handle nonlinear data, high dimensional data and small sample sizes, and exhibit high performance even without parameter tuning [24]. The main disadvantage of RF is the “black box” nature, meaning the resultant models are very difficult to interpret given that they reflect a combination of multiple decision trees. We did not observe an advantage for using feature selection in the RF model. Radiomics tends to produce a much larger feature set necessitating feature reduction in most cases. However, the use of larger parcellation schemes and/or smaller sample sizes may require feature reduction when predicting IDH status from connectomes in patients with gliomas. The addition of age and tumor location to the RF model resulted in a marginal, nonsignificant change in accuracy, consistent with a previous radiomics study showing that age had no effect on model accuracy [7]. The connectome RF model showed a 10% improvement in sensitivity over the clinical RF model (age, tumor location only), a difference that was statistically significant and also likely clinically meaningful to clinicians and patients. However, the clinical model’s performance was excellent and could potentially be improved in future studies such that imaging features are unnecessary. It is also important to note that the highest performance involved a combined connectome and clinical model. Imaging models are inherently more computationally intensive than clinical only models, but it cannot be concluded currently if one approach is robust enough to dismiss the other. All of our models, including the clinical model, require further validation in independent samples.

In addition to novel methods that contribute to ongoing refinement of preoperative IDH status prediction, applying connectomics to this line of research also provides insights regarding the potential neurobiologic effects of IDH mutation. As noted above, our previous study of patients with high grade astrocytoma demonstrated significantly greater connectome disruption and cognitive dysfunction in patients with an IDH wild type tumor compared to those with a mutant tumor, suggesting that differences in lesion momentum and infiltration can affect the entire connectome [6]. The specific mechanisms for these differential effects are currently unclear but may involve known molecular aspects of IDH mutation.

Genes that influence vascular biology, such as VEGF and TGF-β2, are highly expressed in IDH wild type tumors [25]. As a result, these tumors are more angiogenic, show greater cerebral blood volume, higher permeability, and alterations in pericyte and endothelial cell function in comparison to their IDH mutant counterparts [26]. While cerebral vessel disease affects brain structural connectivity [16], it remains to be proven that the vascular phenotype of IDH wild type gliomas contribute to disrupted connectivity differently than that of the mutant variant. IDH mutant tumors are more immunologically quiescent, with fewer tumor infiltrating lymphocytes and less PD-L1 receptors [27], and have reduced expression of genes fundamental to mounting a T-cell response in and around the tumor bed [28]. Inflammatory response is associated with a range of neuropathologies and is thought to contribute to neurodegeneration [29–31]. As activated immune cells are neurotoxic in the absence of any antigen specificity [32, 33], the contribution of a relatively inflammatory microenvironment in IDH wild type gliomas to brain connectivity and integrity warrants further exploration.

D(2)-hydroxyglutarate, a metabolite of IDH mutant cells, exerts an excitatory impact on cultured normal neurons through activation of NMDA receptors [34]. This finding was novel in our understanding of the relationship between glioma cells and the surrounding brain and supports the concept of oncometabolites influencing normal neuronal activity. Mutant IDH reduces the production of NADPH in gliomas [35]. NADPH oxidase has been identified as a major contributor to disease pathology in several neurologic conditions, including amyotrophic lateral sclerosis, Alzheimer’s disease, and Parkinson’s disease. Inhibition pharmacologically of NADPH oxidase enzymes is neuroprotective [36], whether the neurologic insult is degenerative, ischemic, or traumatic [37, 38]. As such, it may be inferred that IDH mutant gliomas, with less NADPH, may have less oxidative toxicity to the surrounding neurons.

In summary, non-invasively predicting IDH status in patients with gliomas from preoperative MRI is a promising line of research with significant clinical relevance. Connectomics is state-of-the art methodology in neuroimaging and neuroscience to date but few if any prior studies have evaluated connectomes to predict IDH genotype. Given the distinct trajectory dependent on IDH status, focusing on molecular profiles rather than histologic grade to determine clinical trials eligibility may become increasingly common, making early definition of IDH subtype imperative. Retrospective data also suggests that patients with IDH mutant gliomas may derive greater benefit from gross total resection than their wild type counterparts [39]. As such, knowledge of IDH subtype pre-operatively may have important implications for preoperative planning, and influence the neurosurgeon’s aggressiveness in removing all visible disease.

We examined four classic machine learning algorithms but there are many others that might apply. We were very vigilant regarding spatial normalization and did not experience any normalization failures, which many studies ignore or fail to mention. However, future connectome studies may require problematic scans to be considered for repair via lesion masking or even exclusion. We focused on connectome features based on prior literature but other connectome properties may be more important. There are also alternative methods for constructing connectomes with respect to node/edge definition, and thresholding, among others, and it is possible that multi-modal neuroimaging models would provide an advantage for prediction. Next generation sequencing for IDH genotype was not available for this retrospective sample and therefore some patients with mutation may have been misidentified. Finally, our connectome, clinical and connectome/clinical classification models require further validation using an independent dataset to determine their values in predicting IDH status relative to computational effort. Despite these limitations, our findings suggest that connectomics is a promising approach for predicting IDH genotype from conventional MRI. Importantly, given the prognostic and biologic information supplied by the connectome, it may serve as a valuable tool to follow therapeutic response through the disease course, offering greater depth of what is occurring with the tumor and surrounding brain than standard MRI sequences and clinical data alone.

## Materials and Methods

### Patient characteristics

We retrospectively identified adult (age 18 or older) patients with histopathologically confirmed WHO grade II–IV gliomas and known IDH genotype from biopsy/resection who were newly diagnosed and first treated at The University of Texas MD Anderson Cancer Center. A total of 234 patients met these criteria and also had an available pre-surgical, T1 MRI acquired at 3 Tesla. Patients were treated during the years of 1996–2018. MRI, demographics, genotype and other clinical data were extracted from the electronic medical record as well as the IRB-approved prospective Department of Neuro-Oncology protocol (PROACTIVE, 2012–0441). IDH status was determined via immunohistochemistry. This study was approved by the MD Anderson Cancer Center Institutional Review Board.

### MRI preprocessing and connectome construction

**Figure 3:**
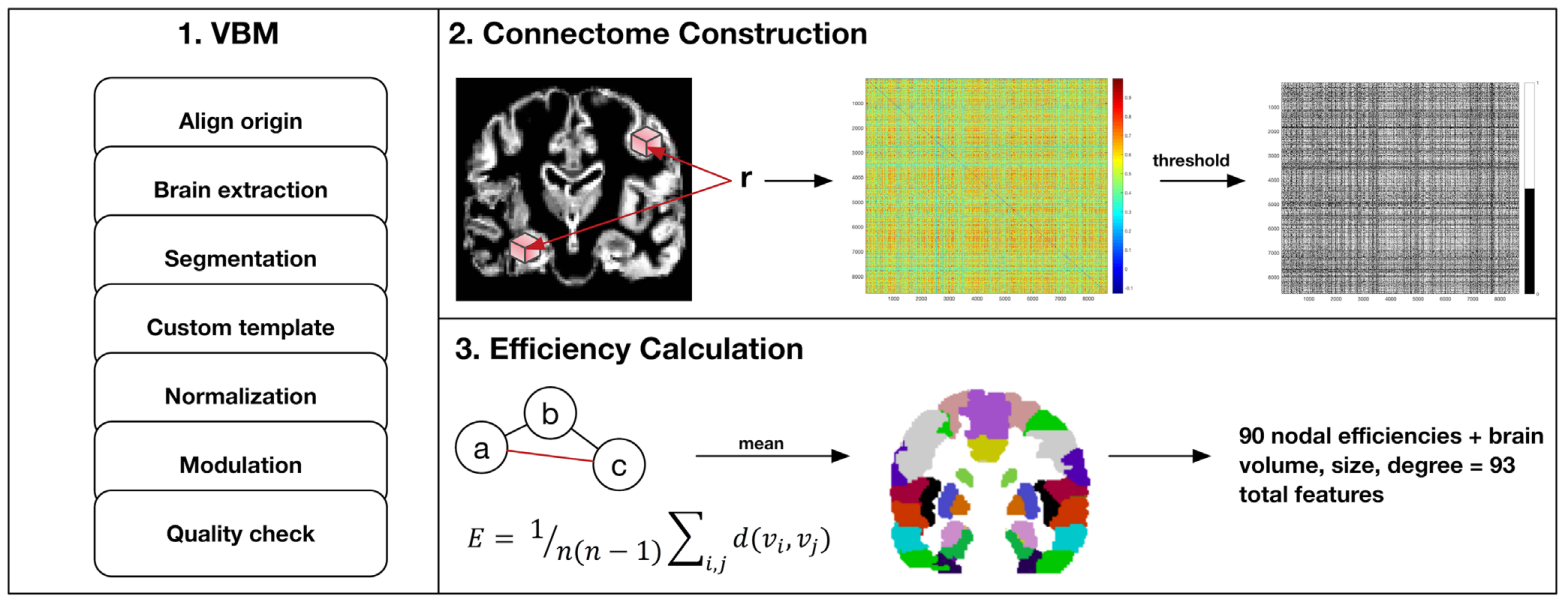
MRI preprocessing and connectome construction steps. First, voxel-based morphometry (VBM) involves standard procedures to extract gray matter volumes including reorientation to anterior and posterior commissure for improved spatial normalization, automated removal of skull, probabilistic segmentation into tissue classes (gray, white, CSF), creation of a sample-specific template via DARTEL, spatial normalization to standard Montreal Neurologic Institute (MNI) space, modulation using jacobian determinant and quality assurance checks. Second, modulated and normalized gray matter volumes were used to construct a connectome map for each patient as the correlation coefficients, r, between voxel values captured by all pairs of 3 **×** 3 **×** 3 voxel cubes (nodes) spanning the entire volume. This correlation, or similarity matrix was then thresholded to remove false positives resulting in a binary matrix where a connection (edge) between two nodes = 1. Third, the binary similarity matrix was submitted to graph theoretical analysis. Efficient information exchange is assumed to follow the shortest path between regions. As illustrated here, the shortest, most efficient path from node a to c is marked in red. Efficiency (*E*) is defined as the average inverse shortest path length across all regions in the network where *n* is the number of nodes and *d(v_i_*,*v_j_)* is the length of the shortest path between nodes *i* and *j*. Efficiencies were averaged across all cubic nodes with MNI coordinates within one of 90 discrete anatomic regions defined by the Automated Anatomical Labeling Atlas (AAL).

Figure 3 summarizes MRI analysis procedures. Gray matter volumes were segmented from pre-surgical, T1-weighted MRI using voxel-based morphometry (VBM) via VBM8 Toolbox and Statistical Parametric Mapping 8 software (Wellcome Trust Centre for Neuroimaging, London, UK). We employed DARTEL, which uses a large deformation framework to preserve topology and employs customized, sample-specific templates resulting in superior image registration, even in lesioned brains, compared to other automated methods [40]. Successful normalization was confirmed using visual and quantitative quality assurance methods [6].

Gray matter covariance networks were constructed for each patient using a similarity-based extraction method [41]. Matrices were then submitted to graph theoretical analysis using Brain Connectivity Toolbox (https://sites.google.com/site/bctnet/) and in-house code (https://github.com/srkesler/bNets.git) implemented in Matlab v2016b (Mathworks, Inc, Natick, MA). We calculated connectome efficiency [42] for each node given that this property is consistently observed to be affected in patients with diffuse glioma [6]. We also computed total brain volume, network size (number of nodes) and degree (number of nodal connections) as these can influence connectome measurements. Network size naturally varies across individuals (mean = 7,158 +/– 43 nodes) so gray matter volumes were collapsed across 90 cortical and subcortical regions [43] to facilitate analyses [6]. Models included all 90 nodal efficiency values plus brain volume, size and degree for a total of 93 features. Random minority oversampling was employed to balance classes.

### Prediction of IDH status

We evaluated the performance of four established machine learning classifiers (Table 2) to predict IDH genotype from pre-surgical MRI connectome features: random forest (RF), support vector machine (SVM) with a polynomial kernel, logistic regression (LR) and neural network (multilayer perceptron - MLP). These algorithms are routinely used in radiomic prediction of IDH status [8–10]. All models were implemented in the Waikato Environment for Knowledge Analysis (Weka) software v3.8.3 (Waikato University, New Zealand), an open source workbench for practical, accessible machine learning applications. Classifier performance was tested using leave-one-out cross-validation and quantified with the area under the receiver operator characteristic curve (AUC). Default Weka tuning parameters were used for simplicity and to increase reproducibility with the exception that number of trees for RF was set to 1000 vs. 100 as the latter is an uncommonly low number of trees in our experience (Table 2). We compared model AUCs using a bootstrapping method implemented in the R Environment for Statistical Computing v3.5.3 (R Foundation, Vienna, Austria), using the “pROC” library.

For the best performing model, we tested whether feature selection would further improve performance. We employed two common strategies, recursive feature elimination (RFE), a type of backward selection, and elastic net regression, a regularization technique that yields a sparse model (tuning parameter alpha = 0.5). Feature selection was nested within the cross-validation loop and the outer loop repeated 50 times. We then calculated the mean AUC across the 50 iterations. RFE was conducted in the “caret” library and elastic net was conducted using the “glmnet” library in R. Finally, we evaluated whether adding age and tumor location (lobe, hemisphere) would improve the best performing model and/or yield a best performing model independently of connectome features.

## _Abbreviations_

IDH: isocitrate dehydrogenase
AUC: area under the curve
RF: random forest
MLP: multilayer perceptron
SVM: support vector machine
LR: linear regression
RFE: recursive feature elimination
VBM: voxel-based morphometry
